# Acromegaly, inflammation and cardiovascular disease: a review

**DOI:** 10.1007/s11154-020-09560-x

**Published:** 2020-05-26

**Authors:** Thalijn L. C. Wolters, Mihai G. Netea, Niels P. Riksen, Adrianus R. M. M. Hermus, Romana T. Netea-Maier

**Affiliations:** 1 Department of Internal Medicine, Radboud University Medical Center Nijmegen, Geert Grooteplein Zuid 10, 6525 GA Nijmegen, The Netherlands; 2 Department for Genomics & Immunoregulation, Life and Medical Sciences Institute (LIMES), University of Bonn, Bonn, Germany

**Keywords:** Inflammation, Insulin-like growth Factor-1, Cardiovascular disease, Acromegaly, Growth hormone, Cytokines

## Abstract

Acromegaly is characterized by Growth Hormone (GH) and Insulin-like Growth Factor 1 (IGF-1) excess. Uncontrolled acromegaly is associated with a strongly increased risk of cardiovascular disease (CVD), and numerous cardiovascular risk factors remain present after remission. GH and IGF-1 have numerous effects on the immune and cardiovascular system. Since endothelial damage and systemic inflammation are strongly linked to the development of CVD, and have been suggested to be present in both controlled as uncontrolled acromegaly, they may explain the presence of both micro- and macrovascular dysfunction in these patients. In addition, these changes seem to be only partially reversible after remission, as illustrated by the often reported presence of endothelial dysfunction and microvascular damage in controlled acromegaly. Previous studies suggest that insulin resistance, oxidative stress, and endothelial dysfunction are involved in the development of CVD in acromegaly. Not surprisingly, these processes are associated with systemic inflammation and respond to GH/IGF-1 normalizing treatment.

## Introduction

Acromegaly is caused by excessive growth hormone (GH) secretion, generally by a pituitary adenoma, and concomitant Insulin-like Growth Factor 1 (IGF-1) excess. [1, 2]. GH and IGF-1 excess exerts many actions on the cardiovascular (CV) system, and CV comorbidities and disease (CVD) risk factors are common, especially in active acromegaly [3–5], but often persist after adequate treatment in patients with controlled disease [6–9]. Circulating IGF-1 inhibits GH secretion via direct negative feedback on the pituitary and also indirectly via stimulating hypothalamic somatostatin secretion [10]. IGF-1 has multiple systemic, autocrine, and paracrine effects, that are specific for the tissues, cellular pathways and metabolic circumstances in which they take place [11]. In addition, the GH and IGF-1/insulin signaling pathways share certain downstream elements, which facilitate the crosstalk between these pathways [11]. The interaction with other hormones and growth factors modulate the effects of IGF-1 [12].

The management of acromegaly and its associated comorbidities has improved over the last decades, resulting in a decreased incidence of macrovascular events. Some more contemporary studies reported the incidence of these macrovascular complications in patients with controlled acromegaly who are treated in expert centers to be nearly comparable to the general population, leading to a nearly restored life expectancy and a mortality risk that is comparable to the general population [8], despite the persistence of CV risk factors.

Basic research has shown that GH/IGF-1 excess has a direct negative impact on multiple components of the system that regulates endothelial function, and contributes to a pro-atherogenic environment via effects on the endothelium. Studies on preclinical and clinical markers of macrovascular damage in acromegaly patients have reported contradictory results, but endothelial and microvascular dysfunction seems to be present in acromegaly patients [13, 14]. The mechanism by which these factors impact on the risk of CVD may be different than in the general population. Despite their unclear significance regarding overt macrovascular damage in acromegaly, cardiovascular derangements represent a therapy challenge and a clinical burden, that require intensive management and negatively impact on QoL [7, 15].

**Figure 1.**
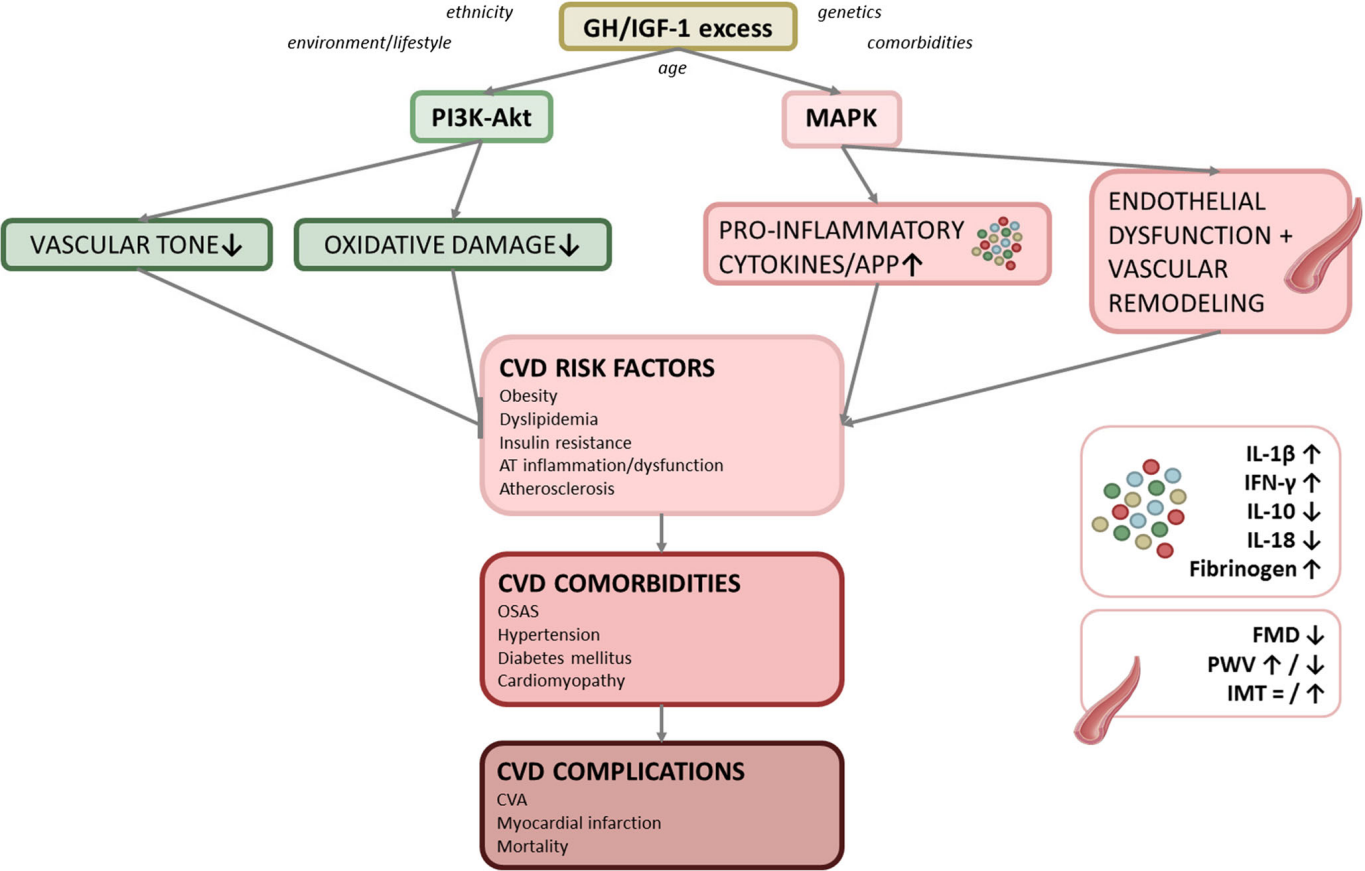
**Schematic overview of the effect of GH/IGF-1 excess on cardiovascular disease-related morbidity.** In Growth Hormone (GH)/Insulin-like Growth Factor 1 (IGF-1) excess, the pro-atherosclerotic mitogen-activated protein kinase (MAPK)-mediated effects are suggested to predominate over the beneficial phosphoinositide 3-kinase (PI3K)-Akt-mediated effects, which renders a negative impact on the cardiovascular system. This balance is also modified by circulating inflammatory, metabolic and endocrine factors, and by age, ethnicity, genetics and environmental modulation (diet, behavior). CVD: cardiovascular disease; AT: adipose tissue; OSAS: obstructive sleep apnea syndrome; IL: interleukin; IFN-γ: interferon gamma; APP: acute phase proteins; FMD: flow-mediated dilatation; PWV: pulse wave velocity; IMT: intima-media thickness; CVA: cerebrovascular accident.

In recent years, numerous publications have confirmed that systemic inflammation is strongly linked to the development of CVD [16–19]. Interestingly, also GH and IGF-1 have been shown to impact on the immune cells and the CV system [20]. Since the prevalence of endothelial dysfunction, metabolic disturbances, and hypertension remains increased in patients with controlled acromegaly compared to the general population, it has been suggested that specific traits of systemic inflammation are induced during GH/IGF-1 excess in active acromegaly and partially persist in controlled acromegaly, which may contribute to the development and persistence of CV comorbidities and hence increase the risk of macrovascular events in these patients (Figure 1) [21–24]. The aim of this review is to summarize and interpret the current knowledge on the relation between acromegaly, systemic inflammation and the pathogenesis of CVD, zooming in on the significance of microvascular inflammation and endothelial dysfunction, and on the underlying pathophysiological mechanisms in the context of acromegaly.

## The relation between GH/IGF-1, the endothelium and CVD

### The relation between GH/IGF-1 and CVD

In large population-based cohorts, IGF-1 concentrations in the normal to high-normal range are associated with a lower prevalence of CVD risk factors, and CVD-related morbidity and mortality [3, 25–27]. In addition, mainly anti-atherogenic and cardiovascular protective effects of IGF-1 administration have been reported [3, 6, 28–30]. Consequently, low and sub-physiological circulating IGF-1 levels are associated with an increased prevalence of CVD risk factors such as insulin resistance, hypertension, and with premature atherosclerosis, overt CVD and CVD-related and all-risk mortality [29, 31–34]. In contrast, supraphysiological IGF-1 levels are reported not to be protective, but rather evoke endothelial dysfunction (ED) and CVD [9, 35–42]. However, studies analyzing IGF-1 levels as an independent risk factor for CVD yielded inconclusive results [12] and therefore provide no indisputable evidence of a direct impact of IGF-1 on the process of atherosclerosis and CVD. Possibly, the associations depend on the influence of GH and IGF-1 on underlying risk factors and processes, such as hypertension, insulin resistance and inflammation, and oxidative stress [43].

Conversely, in populations with an increased risk for CVD (e.g. elderly, patients with hypertension or diabetes), an inverse relation between IGF-1 levels and blood pressure was observed, whereas in healthy populations (with IGF-1 levels in the midrange) a weak or neutral association with blood pressure was demonstrated [29, 33]. Moreover, the association between blood pressure and IGF-1 levels switched from negative to positive in cohorts with high circulating IGF-1 levels (e.g. acromegaly patients) [35]. These seemingly contradictory associations between IGF-1 levels and CVD (risk factors) can be explained by a phenomenon called ‘IGF-1 resistance’, which is observed in dysmetabolic states like obesity, hypertension and chronic kidney failure [44], and is characterized by a diminished maximal response to IGF-1 (with or without additional changes in receptor sensitivity) [45, 46]. Indeed, the expression of IGF-1R is known to be strongly dependent on local and circulating IGF-1 levels [47].

IGF-1 resistance is suggested to be caused by pro-inflammatory cytokines, high IGF-1 concentrations, and hyperglycemia [45, 46] via IGF-1 signaling disturbances or lower IGF-1 bioavailability [48, 49]. On the short-term, the presence of a compensatory protective rise in IGF-1 levels is suggested [35, 44], which is initially sufficient to maintain normal blood pressure and partially preserves endothelial function. However, on the long-term, advancing systemic inflammation and metabolic derangements augment IGF-1 resistance and ED, and suppress GH and consequent IGF-1 secretion [50]. In turn, this causes this compensatory mechanism to fail and leads to the development of overt CVD [35, 44].

The IGF-1 resistance hypothesis is supported by two meta-analyses describing an U-shaped relationship between IGF-1 levels and CVD, overall and CV mortality. Increased CVD risk and mortality was observed in both IGF-1 deficiency (e.g. Growth hormone deficiency (GHD)) and IGF-1 excess (e.g. acromegaly) groups [51, 52], whereas decreased mortality was found for IGF-I levels between 0 and +1 SD, which is suggested to be the optimal range in adults [52].

### Acromegalic cardiomyopathy

The chronic cardiotoxic and remodeling effects of GH/IGF-1 overload results in concentric biventricular hypertrophic cardiomyopathy, which may lead to heart failure [3, 6, 53], especially in patients with insufficiently controlled acromegaly [54, 55]. Acromegaly treatment reverses some of the early stage morphological changes and improves cardiac function, especially when IGF-1 levels were normalized [36, 56–59].

Arrhythmias and cardiac valvular disease are also more common in acromegaly patients compared to the general population [6, 7]. Treatment is reported to decrease the incidence of arrhythmias and prevent further deterioration, but did not reverse established cardiac valve disease [7, 36, 60].

### Coronary artery disease in acromegaly

Reports on the risk of coronary artery disease (CAD) and the presence of coronary calcifications are inconsistent, but the prevalence of clinically apparent CAD is similar in well-controlled acromegaly patients (2.5-12%) compared to the general population [7, 61–64]. Likely, the risk of apparent CAD is a consequence of concomitant traditional CVD risk factors rather than direct effects of GH or IGF-1 [7, 36, 63], and consequently, acromegaly treatment reduces risk of CAD via amelioration of CVD risk factors [65]. However, coronary microvascular dysfunction, which has been associated with the development of macrovascular coronary disease, has been reported in patients with controlled and active acromegaly without overt CAD. The presence of coronary microvascular dysfunction correlated with IGF-1 circulating concentrations and was partially reversed by acromegaly treatment [14].

### Effects of GH and IGF-1 on the endothelium

IGF-1 has also autocrine and paracrine effects on immune cells, endothelial cells (EC), and vascular smooth muscle cells (VSMC) [1, 11, 30]. The endothelium is a key component in the regulation of vascular homeostasis, including the recruitment and invasion of pro-inflammatory cells [30]. The production of nitric oxide (NO) by endothelial NO synthase (eNOS) by EC is crucial for the preservation of endothelial function. However, in the absence of its crucial cofactor (tetrahydrobiopterin; BH4) or substrate (l-arginine), eNOS becomes uncoupled and instead of NO, superoxide is produced. Superoxide is the precursor of most reactive oxygen species (ROS), and therefore, eNOS uncoupling is linked to the induction and progression of endothelial dysfunction and atherogenesis [66]. NO is the primary mediator of endothelium-dependent relaxation, but also inhibits platelet adhesion and aggregation, monocyte adhesion, and VSMC growth, and is thereby a negative regulator of vascular inflammation [13].

Physiological levels of IGF-1 and GH (most likely via induction of IGF-1 production) enhance eNOS expression via induction of phosphoinositide 3-kinase (PI3K)-Akt signaling, which increases NO production and inhibits ROS production, thereby reducing vascular tone and limiting oxidative stress and subsequent damage [3, 12, 13, 26, 28–30, 67]. Moreover, physiological levels of GH are reported to reduce vascular inflammation via suppression of the recruitment and activation of circulating macrophages to the vessel wall [30]. IGF-1 also improves plaque stability via improving VSMC survival, and endothelial repair through effects on endothelial progenitor cells (EPC), enhancement of fibrinolysis, and modulation of the expression of adhesion molecules. On the other hand, IGF-1 has migratory, mitogenic, proliferative and dedifferentiating effects via the induction of the mitogen-activated protein kinase (MAPK) signaling pathway in VSMC [29, 68]. Although GHR are reported to be expressed in VSMC [69] and GH is able to induce the MAPK pathway [70], reports regarding GH inducing the MAPK pathway in VSMC are lacking.

**Figure 2.**
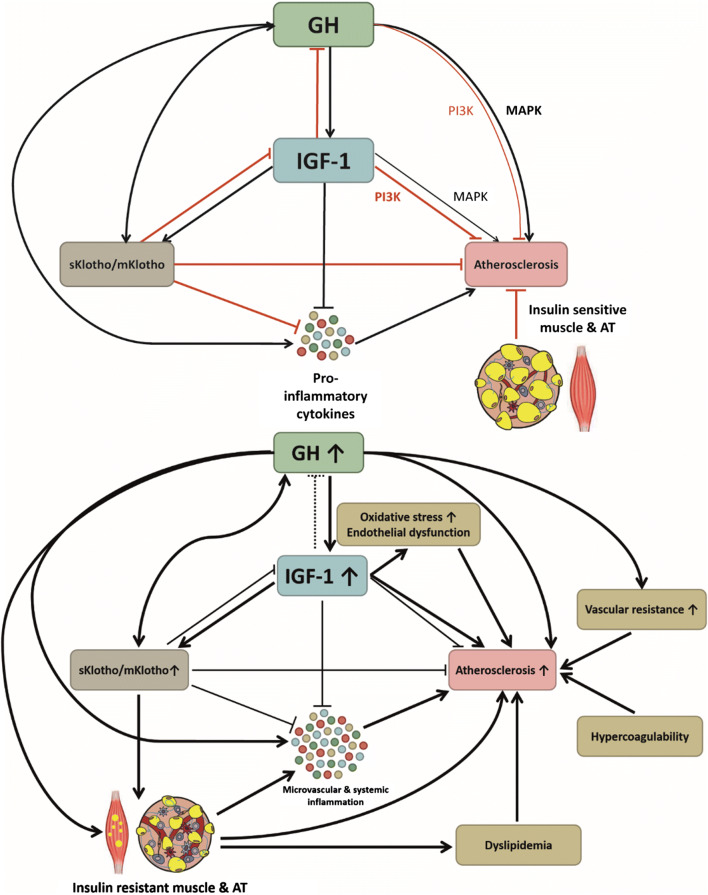
**The relations between GH, IGF-1, Klotho, inflammation and atherosclerosis.** The upper panel depicts the normal physiological situation. Thick arrows depict enhanced effects, whereas thin arrows depict suppressed effects. The balance between GH and IGF-1 is maintained by a negative feedback loop, which also includes Klotho. GH stimulates the production of physiological levels of sKlotho, and sKlotho stimulates GH secretion (direct and via inhibition of the negative feedback loop via IGF-1). A high ratio of soluble (sKlotho) to membrane-bound Klotho (mKlotho) induces inflammation, insulin resistance and atherosclerosis. Healthy muscle and AT inhibit atherosclerosis. Physiological IGF-1 levels have anti-inflammatory and atheroprotective effects via PI3K, whereas GH predominantly has atherogenic effects via MAPK. In the lower panel, the situation in active acromegaly is depicted. In active acromegaly, excessive autonomous GH secretion leads to increased IGF-1 and sKlotho levels, and decreased mKlotho expression. The negative feedback of IGF-1 on GH secretion is disturbed. Both GH and sKlotho induce insulin resistance via ectopic intramuscular adipose tissue (IMAT) deposition and adipose tissue (AT) inflammation. The pro-inflammatory transformed AT and muscle secrete pro-inflammatory cytokines, which stimulate development and persistence of atherosclerosis. The anti-inflammatory and anti-atherogenic effects of mKlotho are overruled by the insulin-antagonizing effects of sKlotho. The potential short-term beneficial effects of GH, Klotho and IGF-1 are overruled by long-term effects of GH and IGF-1 excess: stimulation of pro-inflammatory cytokines, insulin resistance and atherosclerosis. In addition, dyslipidemia, oxidative stress and endothelial dysfunction, increased vascular resistance and hypercoagulability contribute to the development of atherosclerosis.

The net effect of GH/IGF-1 on vasculature depends on the balance between the degree of activation of specific signaling pathways [29, 67, 68, 70] (Figure 1 & 2). IGF-1 expression is increased in atherosclerotic plaques [27], where it stimulates neointima formation and VSMC proliferation, induces chemotactic macrophage migration and activation, and promotes expression of cell adhesion molecules [12].

When the pro-atherosclerotic MAPK-mediated effects predominate over the beneficial PI3K-Akt-mediated effects, GH and IGF-1 negatively impact on the cardiovascular system [71] (Figure 1 & 2). This balance depends on the expression and function of GH/IGF-1, their binding proteins and receptors. In addition, circulating inflammatory, metabolic and endocrine factors, but also age, ethnicity, genetics and environmental modulation (diet, behavior) modulate the effects of GH and IGF-1 [35].

## CVD risk factors in patients with acromegaly

Patients with acromegaly have multiple closely intertwined risk factors for CVD including hypertension, a modified body composition and an adverse metabolic profile characterized by dyslipidemia, DM and/or impaired glucose sensitivity and insulin resistance, and OSAS.

### Hypertension

One third to half of the patients with acromegaly has hypertension [3, 72, 73]. Likely, the hypertrophic effects of IGF-1 and/or GH on the vessel wall, the presence of (O)SAS, and abnormalities in glucose and lipid metabolism are contributing factors [6, 74]. Moreover, GH is reported to increase the total plasma volume and renal sodium reabsorption [72], and cause ED via impaired NO production [4, 36, 73, 75]. Hypertension is only partly reversible with acromegaly treatment [7, 9, 36, 76, 77].

### Metabolic profile and body composition in acromegaly

Active acromegaly is characterized by a decreased fat mass and an increased lean body mass, intermuscular adipose tissue (IMAT) mass and basal metabolism [23, 78, 79]. Despite the lower absolute adipose tissue (AT) mass, the GH-induced dysfunction of visceral AT and increased amount of IMAT likely play an important role in the development and persistence of insulin resistance and impaired glucose metabolism in AT and skeletal muscle in acromegaly patients [23, 78, 80, 81]. After successful treatment of acromegaly, lean mass and basal metabolism decrease and fat mass increases, predominantly in visceral and trunk AT depots [82]. In line with this, AT and skeletal muscle mass are reported to correlate with IGF-1 levels [83]. Whether the properties of the newly acquired AT are similar to those of healthy fat, is unclear.

### Dyslipidemia

Compared to healthy controls, both unchanged [24, 84–86] and pro-atherogenic lipid profiles - characterized by increased concentrations of triglycerides (TG), low-density lipoprotein-cholesterol (LDL-C) and decreased high-density lipoprotein cholesterol (HDL-C) levels - have been reported [21, 22, 65, 87–89] in both active and controlled acromegaly. In controlled acromegaly, lower LDL-C and TG, and higher HDL-C levels have been reported compared to active acromegaly by some studies [83, 90], but others studies reported comparable serum lipid levels [24, 83, 85, 86, 90, 91]. In active acromegaly, pro-atherogenic lipoproteine (a) (Lp(a)) levels were reported to be higher than in controlled acromegaly and healthy controls in some [88, 92, 93], but not all studies [84, 94, 95]. The different acromegaly treatment modalities have distinct effects on lipid levels, although all modalities uniformly resulted in a decrease in Lp(a) levels [76, 84, 92, 93, 96] and an increase in anti-atherogenic APOA1 levels [92, 96].

### Diabetes mellitus

Insulin resistance and diabetes mellitus (DM) are common findings in acromegaly [23]. Especially patients with active acromegaly have higher circulating insulin and glucose levels compared to controls [21, 84, 87, 88, 97]. The relation between IGF-1 excess and disturbances in glucose homeostasis is further supported by the positive association between circulating IGF-1 levels and the prevalence of DM [9].

Dyslipidemia and OSAS are additional risk factors for insulin resistance that are common in acromegaly. Furthermore, altered AT distribution and AT inflammation potentially contribute [23], since systemic inflammation is directly linked to insulin resistance [84, 97]. After treatment of acromegaly, insulin resistance decreased and glucose homeostasis improved, even when IGF-1 normalization was not achieved [81]. However, somatostatin analogues, especially Pasireotide, may deteriorate glucose metabolism [7, 57, 81].

### Obstructive sleep apnea syndrome

(Obstructive) sleep apnea syndrome ((O)SAS) is common with a prevalence of 44-87.5% in active and 35-58% in controlled acromegaly [98, 99]. OSAS is associated with ED, hypertension, systemic inflammation, and dysglycemia; all factors associated with an increased risk of CVD [100]. In general, acromegaly treatment had a positive effect on OSAS, but did not always result in cure [101, 102].

### Preclinical markers of atherosclerosis and CVD in acromegaly

Flow-mediated dilatation (FMD) is a measure of endothelial function and is reported to be lower in active acromegaly compared to matched healthy controls [38, 103, 104]. However, the vasodilator response to nitroglycerine (i.e. endothelial-independent vasodilatation) was similar, which indicates that the difference in FMD indeed displays ED in acromegaly patients [38]. The FMD of patients with controlled acromegaly is between values observed in CV matched or healthy controls and those observed in active acromegaly patients [37, 38, 57, 89, 104], which suggests that ED is only partially reversible after adequate treatment of acromegaly [57, 89, 105]. Interestingly, GHD is also associated with ED [30], which suggests an U-shaped relationship between IGF-1 and endothelial function, where both GH/IGF-1 deficiency and excess induce ED.

Macrovascular stiffening is a later, but still subclinical, stage in atherosclerosis development and can also be a sign of media sclerosis, which are both important processes leading to CVD [106, 107]. PWV can be determined by Pulse Wave Velocity (PWV) analysis. In active acromegaly, PWV is reported to be higher (indicating arterial stiffness) than in healthy controls [37, 86, 108], and PWV decreased after remission [57, 109]. However, others reported no differences between active and controlled acromegaly patients [108], or between patients and matched controls [104]. In a cross-sectional study, we observed lower PWV combined to a lower FMD in a cohort of controlled acromegaly patients with a low prevalence of (strictly controlled) hypertension and DM compared to matched controls [24].

The intima-media thickness (IMT) is a non-invasive marker of early arterial wall alterations associated with atherosclerosis. IMT is reported to be similar [108] or increased [37, 38, 87] when comparing acromegaly patients to healthy controls. Mostly, no differences were observed between patients with active acromegaly, controlled acromegaly and CV matched controls [37, 38, 108], although a recent meta-analysis reported a slightly higher IMT in active compared to controlled acromegaly patients [37].

### **Endothelial dysfunction and microvascular** inflammation in acromegaly

Defects of endothelial function can be considered an early marker of atherosclerosis and cardiovascular dysfunction [110]. Very recently, microvascular inflammation and subsequent endothelial dysfunction have been described as a novel pathophysiological mechanism involved in the development of heart failure with preserved ejection fraction [111].

Interestingly, IGF-1 excess also leads to overexpression of cell adhesion molecules, that possess several pro-inflammatory properties, which is a feature of microvascular inflammation leading to ED. IGF-1 excess is assumed to be a complementary pathogenic factor for ED, which implies that initial endothelial damage (for example by oxidized LDL (oxLDL) or shear stress) is mandatory for the initiation of IGF-1 attenuated endothelial damage via its mitogenic properties [13].

In line with this, reduced levels of NO and eNOS expression [112, 113] and increased levels of markers of oxidative stress have been reported in active acromegaly patients [114, 115]. In addition, eNOS expression and NO levels were inversely correlated with GH/IGF-1 levels [112], whereas oxLDL levels correlated positively [115]. It has been suggested that IGF-1 excess decreases NO production via induction of insulin resistance, but this relation has not been observed in all studies [116].

Increased ROS formation causes oxidative stress by decreasing NO availability and subsequent increased levels of oxLDL, which promotes chemotaxis and activation of leukocytes (especially monocytes). Monocyte-derived macrophages that invaded the vascular wall are stimulated to form foam cells, which further fuels vascular wall inflammation and damage. Indeed, Boero *et al.* reported increased levels of oxLDL in active acromegaly patients and increased oxidative stress, which was also suggested by others [113, 115, 117], although increased oxidative stress or a reduced oxidative capacity was not observed in all studies [118].

Multiple studies have reported that measures of microvascular function are impaired in active acromegaly patients [14, 108, 116]. Next to functional consequences, IGF-1 also is reported to impact on microvascular structure. The most observed change observed in acromegaly is microvascular wall hypertrophy. These structural changes could also impact on microvascular function. In addition, both microvascular structure and function improve with disease control, but remain impaired in patients with controlled disease [14, 108, 116, 119, 120]. Microvascular dysfunction correlates with IGF-1 levels [14, 116]. Interestingly, microvascular dysfunction has also been observed in GHD, and improves after GH substitution therapy, providing additional evidence for the presence of a U-shaped relation between IGF-1 and endothelial damage [13, 67].

**Figure 3.**
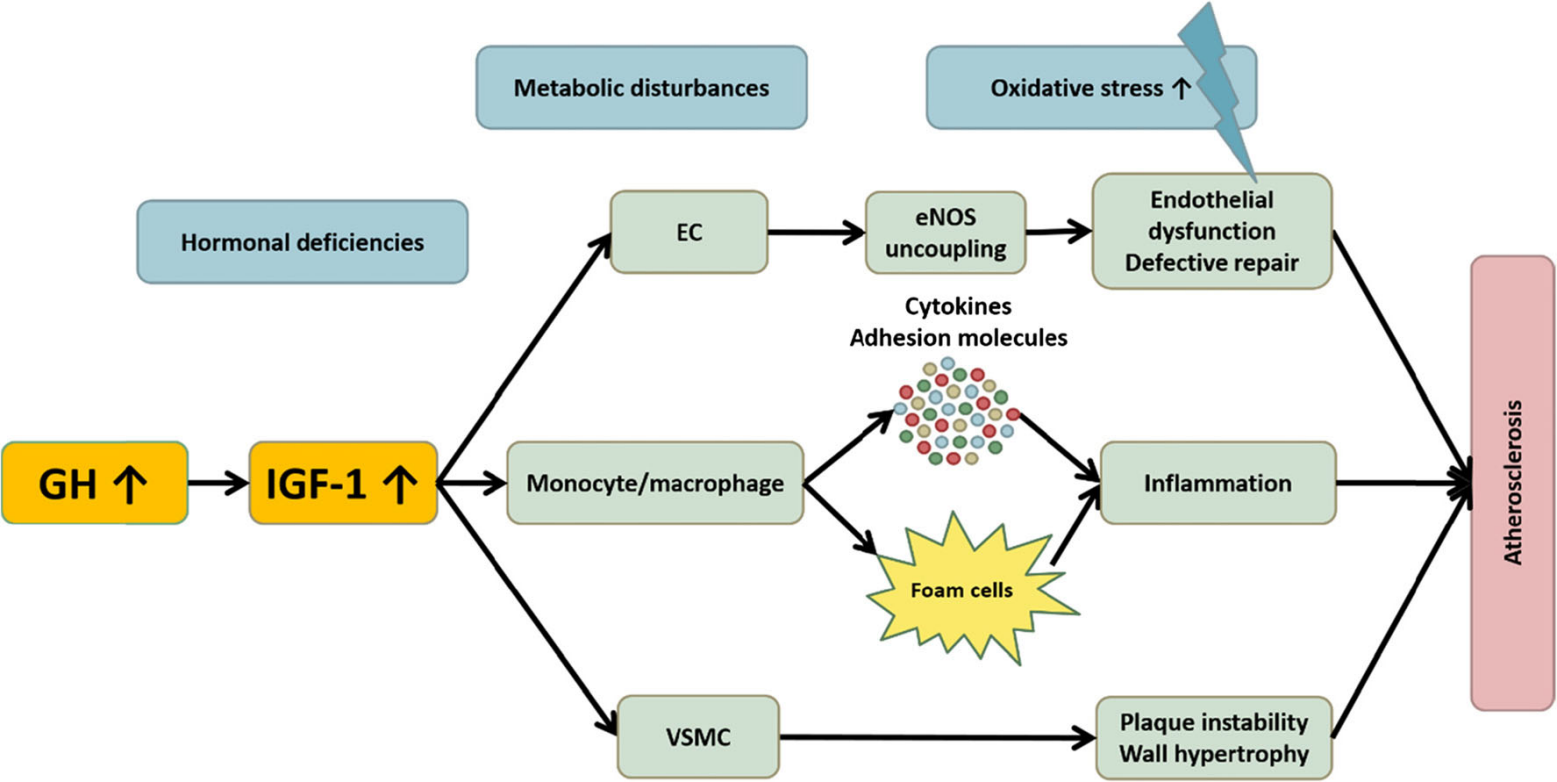
GH/IGF-1 excess is suggested to cause endothelial damage via increased oxidative stress, microvascular inflammation and VSMC proliferation. Additional contributing factors are concomitant hormonal deficiencies and metabolic disturbances.

Taken together, microvascular inflammation and early atherosclerotic changes, especially ED, are more prevalent in acromegaly compared to healthy controls, and are only partly reversible with disease control. Additional factors present that may contribute to ED and CV disturbances in acromegaly patients are CV comorbidities as hypertension and metabolic disturbances, concomitant hormonal disturbances as hypogonadism, increased levels of pro-inflammatory cytokines and expression of adhesion molecules, disturbed endothelial repair mechanisms and vascular alterations caused by proliferation of VSMC (Figure 3). However, structural changes of major vessels have not been proven to be more prevalent in well-controlled acromegaly patients than in controls.

## Inflammation in the pathogenesis of atherosclerosis and CVD

In recent years it has become apparent that CVD is strongly associated with low-grade systemic inflammation [19, 121]. Low-grade vascular wall inflammation is an important player in the initiation and progression of atherosclerosis. Endothelial cell activation trigger immune cell trafficking into the vascular wall via expression of vascular cell adhesion molecules (CAMs)). These molecules attract platelets and monocytes, which migrate into the subendothelial layer and promote local inflammation. Once in the plaque, monocyte-derived macrophages become foam cells, which form a fatty streak in the arterial intimal layer and fuel local inflammation by the production of cytokines, such as interleukin (IL)-6, tumor necrosis factor (TNF)-α, and chemokines as IL-8 and Monocyte Chemoattractant Protein (MCP)-1 [18, 122–124]. In reaction, VSMC are stimulated to proliferate and migrate into the intima, which thickens and bulges out into the arterial lumen, and may impair blood flow. VSMC synthesize collagen to form a fibrous layer that encloses the lipid core. Eventually the plaque consists of a fibrous cap, an underlying necrotic core, and many inflammatory cells. Activated macrophages produce matrix metalloproteinases (MMP) that contribute to destabilization of the plaque [18, 124]. The more advanced the plaque gets, the more vulnerable it is to rupture. Plaque rupture or plaque erosion induces platelet attachment and thrombosis, which may acutely obstruct the vessel, or cause downstream embolization by plaque particles, with subsequent ischemia in adjacent tissues [125, 126].

Despite the rising amount of knowledge on the relation between inflammation and CVD, the unresolving character of the low-grade inflammation that drives atherosclerosis remains poorly understood. In recent years, it became apparent that innate immune cells can develop long-term functional reprogramming characterized by hyperresponsiveness: trained immunity [127]. Short-term exposure to stimuli of both bacterial and non-bacterial origin can induce a long-term pro-inflammatory phenotype of monocyte-derived macrophages, both ex vivo [128, 129] as in vivo [130]. Indeed, in patients with risk factors for atherosclerosis or with established atherosclerosis, circulating monocytes have a pro-inflammatory phenotype, characterized by an increased cytokine production capacity [19, 131–133]. It is suggested that this concept may also apply to acromegaly [118, 134–137], since GH and IGF-1 have repeatedly been reported to impact on the immune system [20, 138, 139]. Moreover, circulating immune cells express GH and IGF-1 receptors [138, 140, 141], and macrophages and lymphocytes can produce IGF-1 [20, 142, 143].

## GH, IGF-1 and GH/IGF-1 disturbances as modulators of inflammation

### Effects of GH, IGF-1 and GH/IGF-1 disturbances on ex vivo cytokine production

Several studies have reported that GH and IGF-1 influence ex vivo cytokine production in circulating immune cells, while others found no effects. The more conflicting results concern particularly the effects of GH. The interest in studying the inflammatory effects of GH and IGF-1 initiated from the observation that high-dose GH treatment in critically ill patients, which was aimed to overcome their catabolic state, was associated with an increased mortality. It was hypothesized that supraphysiological dosages of GH increase pro-inflammatory cytokine production, either via direct effects on immune cells or via increased production of IGF-1, which might have contributed to the increased mortality in this context. Indeed, ex vivo stimulation with 200-300 ng/ml GH resulted in an increased secretion of IL-1β and interferon (IFN)-γ & IL-1β, in human macrophages and murine peritoneal macrophages respectively [135, 144]. However, stimulation with GH up to a very high concentration of 10.000 ng/ml had no stimulatory effects on TNF-α secretion, and even decreased TNF-α production in peritoneal macrophages. Interestingly, increased production of TNF-α mRNA expression and secretion was observed in human monocytes and murine peritoneal macrophages after stimulation with IGF-1 in the same experimental setting [47]. In another study, ex vivo stimulation of human peripheral blood mononuclear cells (PBMCs) with 100 ng/ml GH, which was expected to elicit a maximal response, did not affect the production of TNF-α, IL-6 and IFN-γ, either in absence or presence of co-stimulation with the Toll-like Receptor (TLR)-ligand Lipopolysaccharide (LPS) [145]. However, co-stimulation with GH and LPS induced IL-6 and TNF-α expression and secretion in whole blood (WB) [146]. In addition, co-stimulation with LPS and GH inhibited TNF-α secretion in mononuclear cells [147]. However, in PBMC from healthy donors such an effect was not observed [145, 148]. These conflicting results may be explained by the presence of a bell-shaped dose-response curve for GH [145]. The maximal effect on pro-inflammatory cytokine production has been observed with a dose of around 300 ng/ml in aforementioned studies. Another explanation for these discrepant results is the time that is required to elicit the maximal effect on cytokine production, since the changed TNF-α production was observed after 1 and 6 hours of culture. However, after 24 hours of culture, very weak or negligible effects were observed. Indeed, negative studies only measured cytokine production after 24 hours of culture. Last, the effects of GH stimulation are likely to be cell-specific, since receptor expression and intracellular signaling pathways differ between cell populations [149, 150]. ​

With respect to IGF-1, stimulation of murine monocytes or peritoneal macrophages with 1-1000 ng/ml IGF-1 is reported to induce TNF-α secretion in one study [47], and increased IL-1β and reduced IL-1Ra secretion in another [135]. The highest response was observed with a dose of 100 ng/ml IGF-1 [47]. Our group did not observe a significant effect of stimulation with 50-5000 ng/ml IGF-1 in PBMCs from healthy volunteers [148]. However, co-stimulation with IGF-1 (50-5000 ng/ml) and a TLR-ligand showed a linear dose-response relation and induced IL-6, IL-10, IFN-γ and TNF-α secretion in human PBMC [148, 151, 152] and monocytes [47]. In murine mast cells, co-stimulation with LPS induced IL-6 and TNF-α and reduced IL-1β protein secretion [153]. Also in whole blood from healthy volunteers, co-stimulation increased IL-6 secretion and non-significantly decreased IL-1β secretion [148]. Others found that the combination of IGF-1 and T-cell-activating PHA (phytohaemagglutinine) modestly decreased the secretion of pro-inflammatory IFN-γ, whereas it stimulated the secretion of anti-inflammatory IL-10 in human mononuclear cells. However, there were also small stimulatory effects on secretion of monocyte-derived cytokines TNF-α, IL-6, IL-8 and IL-1β [154]. Interestingly, stimulation with LPS resulted in higher production of IL-6 and TNF-α in monocytes obtained from adult GHD patients compared to controls [155]. GH substitution therapy inhibited both in vivo and ex vivo cytokine production, although not towards normal levels in all patients [138, 155].Treatment of children with severe burns with IV IGF-1/IGFBP3 for one week, which led to a fourfold increase in circulating IGF-1 levels, increased IFN-γ production and induced a Th1 cytokine response in WB, whereas the Th2 (IL-4, IL-10) response was suppressed [156].

To summarize, both GH and IGF-1 seem to impact on ex vivo cytokine production, but mainly when acting as a co-stimulus to stronger inflammatory stimuli. For GH, a bell-shaped dose-response relation was observed, in which both low and extremely high doses inhibited pro-inflammatory cytokine production or rendered only a minor stimulatory effect. For IGF-1, a linear dose-effect relation was observed, and IGF-1 predominantly induced a Th1 cytokine response (IFN-γ, IL-2). The anti-inflammatory IL-10 response that is observed after short-term, but not longer-term, stimulation is suggested to be a compensatory mechanism for the pro-inflammatory effects of GH and IGF-1 [154].

Also in whole blood obtained from patients with acromegaly, ex-vivo stimulation with TLR-ligands increased IFN-γ and lowered IL-10 secretion. In patients with active acromegaly, higher production of IL-1β compared to controlled patients was observed, next to higher production of IL-1Ra compared to controls. There were no differences between controlled patients and controls. No significant effects were found regarding production of the monocyte-derived cytokines IL-6 and TNF-α, although IGF-1 levels correlated with IL-6, IL-1β, IL-1Ra and IFN-γ secretion [24].

Importantly, long-term endogenous exposure to supraphysiological levels of GH and/or IGF-1 may affect ex-vivo stimulated cytokine production in a different way than shorter-term ex vivo exposure. Exposure to IGF-1, but not GH, induced trained immunity ex vivo and in vivo [157, 158], which is likely to account for the augmented ex vivo stimulated cytokine production from monocytes obtained from acromegaly patients, but also in cells obtained from healthy volunteers that are trained with IGF-1 ex vivo. However, the in vivo effects of GH and IGF-1 are probably more complex since interfering factors likely play a role. For example, pro-inflammatory cytokines are also known to induce GH resistance [138, 159–161], which hampers GH-mediated actions and IGF-1 secretion, and therefore also impact on cytokine production.

The pro-inflammatory phenotype that results in the increased ex vivo cytokine production that is observed when stimulating cells obtained from patients with GHD and acromegaly must be induced in vivo. Whereas the ex vivo dose-response curve following IGF-1 stimulation is linear, mononuclear cells obtained from patients with GHD and acromegaly both display a pro-inflammatory phenotype. Therefore, GHD-related changes in metabolic profile, receptor expression and other factors inducing systemic inflammation in these patients likely contribute to the pro-inflammatory phenotype. However, these changes are at least indirectly related to GH/IGF-1 deficiency, since GH substitution therapy is reported to reserve the pro-inflammatory phenotype [155, 162, 163].

### Circulating cytokines and acute phase proteins in acromegaly

Levels of circulating markers of inflammation are the net result of the production of these markers in all involved tissues, and their subsequent clearance.

In acromegaly patients, reports on circulating inflammatory markers are conflicting. Circulating IL-6 concentrations have been reported to be similar in healthy controls and patients with either active or controlled acromegaly [24, 84, 135, 136, 162, 164]. However, IL-6 concentrations were lower in patients with active disease compared to those with controlled disease [91, 162], and were inversely correlated with IGF-1 and GH levels [162]. TNF-α concentrations were reported to be similar [165] or higher [136], IL-1 concentrations were similar [165] or not significantly higher [136], and IL-8 levels were higher in patients with active acromegaly compared to controls [136]. On the contrary, Ueland *et al.* reported increased circulating IL-1β concentrations with decreased anti-inflammatory IL-1Ra concentrations in patients with active acromegaly, suggesting enhanced IL-1 activity, although IL-1β and IL-1Ra mRNA expression in PBMCs from these patients was similar to controls. IL-1Ra, but not IL-1β concentrations or the IL-1β/IL-1Ra ratio, were inversely correlated with GH levels, IGF-1 levels, and total body fat mass [135].

In analogy, GHD patients displayed a pro-inflammatory phenotype with similar or increased circulating levels of IL-6 and TNF-α when compared to healthy controls; the pro-inflammatory tendency decreased or normalized after GH replacement therapy [155, 162, 163].

Patients with active acromegaly are characterized by an increased pro-inflammatory activity of both the adipocytes and adipose tissue (AT) macrophages [23, 166], despite a decreased AT mass. Since AT macrophages in healthy AT are an important source of anti-inflammatory IL-1Ra and also pro-inflammatory cytokines [166], changed AT composition and/or activity may result in the lower IL-1Ra levels and higher circulating pro-inflammatory cytokine levels that characterize patients with active acromegaly. This theory is supported by reports that ex vivo stimulation of adipocytes with GH increased inflammatory adipokine production, and that AT depots of mice with GH excess contained increased amounts of inflammatory cells [164, 167, 168].

Differences in AT mass might also explain the relatively higher circulating IL-6 levels in patients with GHD and controlled acromegaly (who are characterized by an increased AT mass) compared to patients with active acromegaly (who are characterized by a decreased AT mass) [50, 91, 162].Those patients are both characterized by an increased and pro-inflammatory transformed VAT mass, which is an important source of pro-inflammatory IL-6 [23, 84]. In addition, metabolic disturbances are common in both GHD and acromegaly [6], and may enhance systemic inflammation as was demonstrated by higher levels of circulating pro-inflammatory cytokines and a pro-inflammatory phenotype of mononuclear cells.

Pro-inflammatory cytokines stimulate the secretion of acute phase proteins (APPs) that participate in the inflammatory cascade. Class 1 APPs, such as CRP, are mainly induced by IL-1β, alone or in combination with IL-6. Class 2 includes fibrinogen, and mainly responds to IL-6, but not IL-1β [50, 169]. Increased CRP and fibrinogen levels are both associated with CVD [19, 122, 170–172].

Reports in non-acromegalic subjects suggest that long-term (e.g. several weeks) exposure to supraphysiological levels of GH results in increased inflammatory activity. On the contrary, exposure to physiological levels (which are aimed when treating GHD) or short-term exposure of non-GHD patients to supraphysiological GH dosages has neutral or anti-inflammatory effects [50, 67, 145], which are suggested to be mediated via reduction of oxidative stress via induction of NO synthesis [67].

Surprisingly, and opposite to the aforementioned cytokine patterns, patients with active acromegaly are reported to have lower CRP levels compared to healthy controls [21, 22, 84, 162] and controlled acromegaly patients [83, 88] in most, but not all studies [85, 89, 95, 136]. CRP levels increase following IGF-1 lowering therapy [76, 84, 91, 92], and controlled acromegaly patients display similar levels as healthy controls [85, 89, 91, 95].

**Figure 4.**
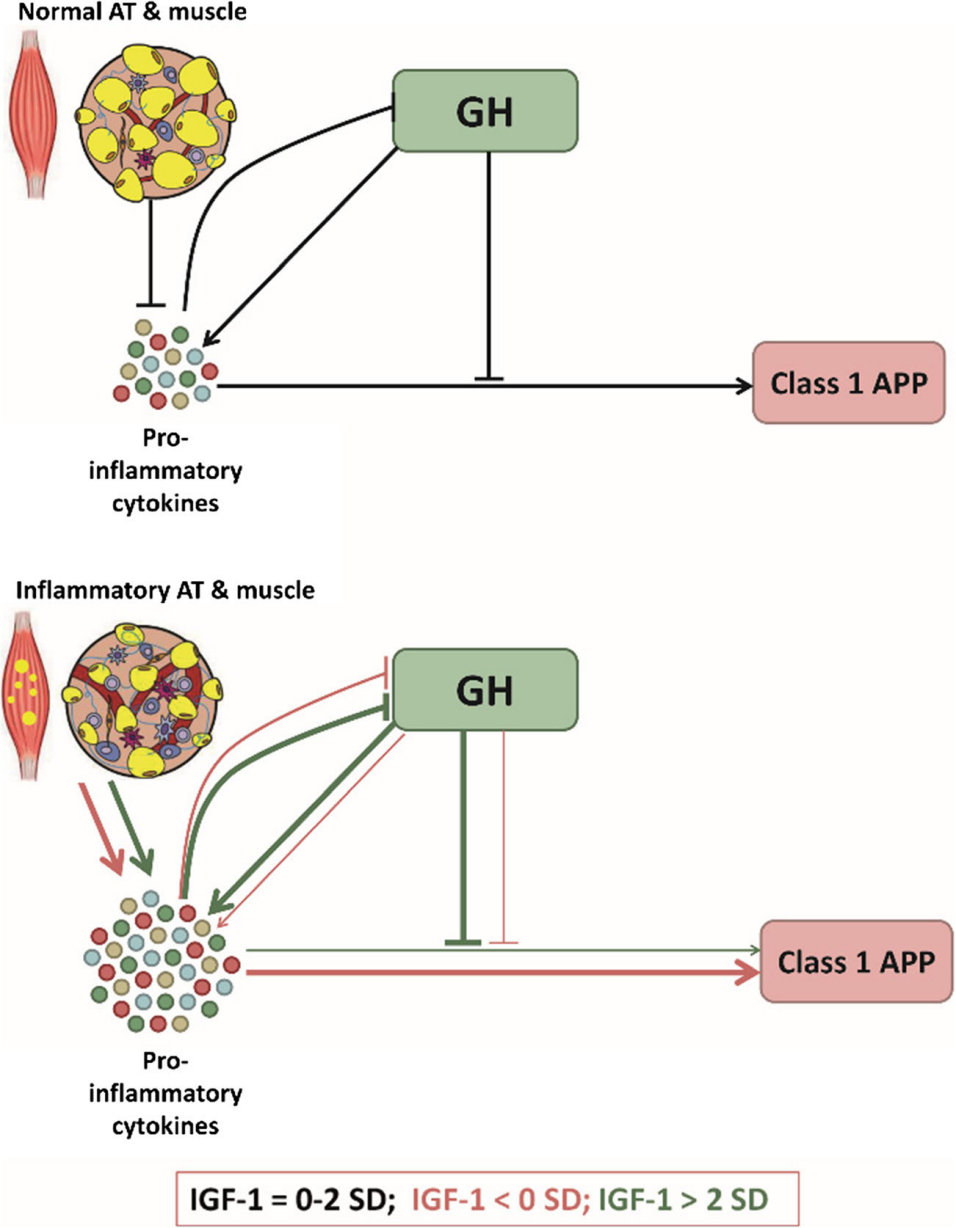
**The relation between GH, IGF-1 and class I acute phase proteins (APP).** In the upper panel, the situation for normal to high-normal (physiological) IGF-1 levels is depicted with black arrows. There is a balance between GH-induced pro-inflammatory cytokine production and APP. Healthy muscle and adipose tissue (AT) inhibit inflammation. In the lower panel, the situation in GH/IGF-1 disturbances is depicted. The pink arrows display the situation in states of low to very low IGF-1 levels, where inflammation in AT induces pro-inflammatory cytokine production and the inhibitory effects of GH on class I APP are attenuated, rendering high levels of both pro-inflammatory cytokines and class I APP.The green arrows display the situation in active acromegaly, where both GH excess and pro-inflammatory transformed AT and muscle induce pro-inflammatory cytokine production, but GH also suppresses class I APP production, rendering higher levels of pro-inflammatory cytokines combined to lower levels of class I APP.

The inverse relation between pro-inflammatory cytokines and class 1 APP in acromegaly may be caused by effects of GH and/or IGF-1 on the interaction between cytokines and APPs (Figure 4). Receptors for GH and IL-6 both belong to the class I cytokine receptors: they display structural similarities and share intracellular signaling pathways [50, 160, 161]. Therefore, interactions as cross-talk and antagonism are possible. In vitro, GH induces suppressor of cytokine signaling (SOCS) 3 expression, which inhibits IL-6 and IL-1β-induced APP production [50, 84, 169]. Also, GH and IGF-1 seem to have suppressive effects on IL-1β production, which may further attenuate class I APP production [50, 153]. The other way around, pro-inflammatory cytokines are reported to stimulate the SOCS family, of which SOCS2 is reported to inhibit GH activity (inducing GH resistance) [67].

Treatment of acromegaly normalizes the disturbed cytokine-APP signaling, leading to normalization of APP levels [87]. These findings suggest a reciprocal relation between GH/IGF-1 and pro-inflammatory cytokines, and imply that class I APP are an unsuitable marker of low-grade vascular inflammation in acromegaly [84].

On the contrary, GHD patients displayed increased circulating CRP concentrations in comparison to healthy controls, and CRP normalized after GH replacement therapy [162, 163]. Probably, the abovementioned mechanism also applies to GHD, since class I APP production is not suppressed by GH-induced SOCS3 expression.

Next to APP and cytokines, numerous other circulating inflammatory and vascular markers have been investigated in patients with acromegaly. However, inconsistent results have been reported for the majority of these markers. An overview of the current knowledge regarding those markers in patients with acromegaly is depicted in Table 1.

**Table 1:** Overview of circulating cardiovascular markers and adipokines

**Marker**	**Patients vs. controls**	**AA vs. CA**	**Effect of acromegaly treatment**	**Effect on CVD**
*Klotho*	AA > Co [173] CA = Co [173]	AA > CA [173]	Decrease [173]	**+**
*FGF-21*	AA + CA> Co [174] AA = CA = Co [175]	AA = CA [174, 175]		**-**
*Il-18*	AA = Co [24] CA < Co	AA > CA [24]		**-**
*VCAM-1*	AA + CA> Co [85] AA = Co [22] AA = Co; CA< Co [24]	AA > CA [24] AA = CA [85]		**+**
*ICAM-1*	AA + CA> Co [85]	AA = CA [85]		**+**
*Homocysteine*	AA = Co [84] AA = CA= Co [85, 88, 176]	AA = CA [84, 85, 88, 176] AA < CA [76, 83]	No effect [84, 177] Increase [76]	**+**
*Endothelin-1*	AA > Co [21, 22, 176]	AA > CA [21, 22, 176]	Decrease [21, 22, 176]	**+**
*E-selectine*	AA > Co; CA= Co [85] AA = CA= Co [24]	AA > CA [85, 178] AA = CA [91]	Decrease after EETA [178]	**+**
*P-selectine*	AA > Co; CA> Co [89]	AA > CA [89]		**+**
*MMP-2*	AA > Co; CA= Co [179]	AA > CA [137, 179]	Decrease [179]	**+**
*MMP-9*	AA = CA= Co [179]	AA = CA [137, 179]	No change [179]	**+**
*MCP-1*		AA > CA [167]	Decrease	**+**
*Angiogenin*	AA > Co [180]			**+**
*VEGF*	Total VGEF: AA= Co [179–181] VEGF-D: AA> Co [180] VEGF-C: AA= Co [180]	AA > CA [179] AA = CA [167]	Decrease after PEGV [179] No change for PEGV/SSA, decrease after EETA [167]	**+**
*Fibrinogen*	AA + CA > Co [87, 92, 182–184] AA > Co [88, 95, 185–189] CA > Co [187] AA = CA = Co [190]	AA > CA [88, 92, 95, 184, 188, 191] CA > CA with GHD [192] AA = CA [87, 90, 183, 190, 192]	Decrease [92, 95, 184, 188, 191]	**+**
*PAI-1*	AA > Co [92, 185] AA = Co [184, 189]	AA > CA [92] AA = CA [184]	Decrease [92, 184] No change [184]	**+**
*Adiponectin*	AA > Co [193–195] AA = Co [196, 197] CA = Co [194] AA < Co [198] AA + CA < Co [199]	AA > CA [194] AA = CA [91, 199, 200] AA < CA [198]	No change in total group [96, 167] Decrease after EETA [167] Increase after SSA, EETA, PEGV [167, 198, 201]	**-**
*Leptin*	AA = Co [202–206] AA < Co [164, 193, 196, 207–212]	AA = CA [213] AA < CA [76, 83]	Increase after SSA/EETA [208, 214–216] Decrease after single dose Lanreotide [210] No change [96]	**+**
*Ghrelin*	AA = Co (trend A A< Co) [205, 209, 217–219] CA = Co [220] AA < Co [196, 206, 220–222]	AA = CA [209, 219] AA < CA [76, 213, 220, 222–224]	Increase after remission/EETA/PEGV [76, 209, 220, 223, 224] Decrease after SSA [213, 217, 220, 223, 224] No change in ratio acetylated/non-acetylated ghrelin [224]	**-**
*Visfatin*	AA > Co [164] AA = CA = Co [199]	AA > CA [200] AA = CA [199]		**+**
*Resistin*	AA = Co [193] AA < Co [203]	AA > CA [200]		**+**
*Omentin*		AA < CA [96]	Increase	**-**
*Vaspin*		AA > CA	Decrease	**-**

CVD: cardiovascular disease; AA: Active acromegaly; CA: controlled acromegaly; Co: controls; SSA: somatostatin analogue; PEGV: Pegvisomant; EETA: endoscopic endonasal transsphenoidal adenomectomy; FGF-21: Fibroblast Growth Factor -21; IlIL-18: Interleukin 18; VCAM-1: Vascular Cell Adhesion Protein 1; ICAM-1: Intercellular Adhesion Protein 1; MMP-2: Matrix Metalloproteinase-2; MMP-9: Matrix Metalloproteinase-9; MCP-1: Monocyte Chemoattractant Protein 1; VEGF: Vascular Endothelial Growth Factor; PAI-1: Plasminogen Activator Inhibitor 1

Summarizing, it can be stated that active acromegaly is characterized by an adverse circulating cardiovascular and inflammatory marker profile (Figure 1). Most markers improve after treatment, although controlled acromegaly patients still display an unfavourable profile compared to controls. The overall finding that IGF-1 normalization is associated with CVD risk reduction (but not normalization) [14, 65, 183], emphasizes that obtaining complete remission is important in the optimal management of CVD risk factors.

#### Klotho

α-Klotho is a circulating marker of particular interest with respect to the relationship between IGF-1, inflammation and atherosclerosis. It stimulates adipogenesis [225] and maintains endothelial function via enhancing anti-oxidative activity [226]. In addition, soluble Klotho (sKlotho) increases insulin secretion and attenuates the development of DM type 2 by improving β-cell function and survival [226]. The membrane-bound form of Klotho (mKlotho) is a co-factor for the interaction between fibroblast growth factor-23 (FGF-23) and its receptor (FGF-23R), and modulates its role in renal calcium and phosphate homeostasis [225].

Insulin and IGF-1 induce mKlotho’s cleavage via ADAM17, rendering soluble Klotho (sKlotho). Lower amounts of mKlotho impair FGF-23 signaling, leading to hyperphosphatemia, which impairs endothelial function. Klotho is cleaved by ADAMTS17, which also cleaves the GHR, yielding a soluble GHR which acts as a GHBP. Subsequently, Klotho inhibits the IGF-1 pathway, enhances GH secretion [226], and stimulates sKlotho secretion via a positive feedback loop [226]. In addition, sKlotho interacts synergistically with basic FGF, which also induces pituitary GH secretion [226].

Administration of sKlotho attenuated inflammatory cytokine production and adhesion molecule expression in murine models with Klotho deficiency [227, 228]. Also in humans, lower sKlotho levels are associated with atherosclerosis and inflammation [226, 229]. In active, but not in controlled acromegaly, high levels of sKlotho and FGF-23 have been reported compared to controls [173]. After remission, sKlotho levels rapidly normalized [173]. The potentially beneficial effects of the high sKlotho levels on endothelial function and inflammation seem to be outweighed by negative effects of hyperphosphataemia and other disturbances related to GH/IGF-1 excess, and are insufficient to reverse the increased cardiovascular risk in acromegaly.

## Perspective on the role of inflammation in the pathogenesis of CVD in patients with acromegaly

From the previous section it is clear that CVD risk factors, microvascular dysfunction and systemic inflammation are more prevalent in acromegaly patients, especially those with active disease, but also persist in those with controlled disease. This stresses that management of CVD needs to be improved. It is likely that GH and IGF-1 excess has a negative impact on the CV and immune system, which may contribute to the pathogenesis of CVD in patients with acromegaly. However, studies on the role of the immune system in the development of CVD in acromegaly are scarce, and both increased as well as unaltered inflammatory and cardiovascular markers have been reported [21, 22, 134, 136].

The net effect of acromegaly on inflammation and CVD seems to be influenced by disease control (GH/IGF-1 levels), concomitant metabolic disturbances, hormonal deficiencies, age and gender [104, 105], which illustrates the complex mechanisms and interactions with GH/IGF-1 and other factors. In addition, the effects of GH and IGF-1 seem to be time- and dose-dependent: in states with either subphysiological or prolonged exposure to supraphysiological GH/IGF-1 concentrations, a pro-inflammatory state and atherosclerotic changes were observed (Figure 3). On the contrary, normal to high-normal circulating concentrations or short-term exposure to mildly increased GH/IGF-1 levels exert beneficial effects (Figure 2). However, on the longer term, these beneficial effects are outweighed by GH-induced mitogenic effects on VSMC and contra-regulatory mechanisms as IGF-1 resistance.

Although some anti-atherogenic effects of IGF-1 on the endothelium have also been observed in acromegaly, as demonstrated by the lower than expected extent of atherosclerosis (i.e. lower IMT, based on risk factors, compared to controls) in active acromegaly patients [230], the pro-atherosclerotic and pro-inflammatory effects seemed to predominate on the longer term [108].

The attenuation of beneficial effects of GH and IGF-1 on the long-term may be explained by pro-inflammatory cytokines, which were reported to induce resistance to IGF-1 [45], but also reduce IGF-1 production and other potentially beneficial GH-mediated effects [231]. Although GH has been reported to inhibit pro-inflammatory cytokine production, this action seemed to be counterbalanced by the increased cytokine production in pro-inflammatory transformed AT. The lower amount of AT in active acromegaly seems insufficient to counteract the pro-inflammatory changes in the remaining AT, which also produces high amounts of unfavorable adipokines, and induces insulin resistance. The additional negative effects of GH excess on glucose homeostasis may further restrict the potential beneficial effects of IGF-1, which is supported by the observation that IGF-1 preferentially induced the pro-atherogenic MAPK pathway over the protective PI3K-Akt pathway in hyperglycemic conditions [232].

GH/IGF-1 and AT status impact on abovementioned effects since patients who develop GHD after cure of acromegaly displayed higher inflammatory markers (fibrinogen, CRP) and had an unfavorable body composition with increased AT mass compared to cured patients without GHD [192].

Besides the importance of exposure time and dose, additional factors are suggested to impact on systemic inflammation and metabolic dysregulation in acromegaly, such as the presence of OSAS. In addition, Klotho might be a link between GH/IGF-1, inflammation and metabolic dysregulation in acromegaly, since Klotho impacts on all these components (Figure 2). Moreover, a hypercoagulable state was reported in active acromegaly [93, 185, 186]. Since inflammation activates coagulation and coagulation factors in return enhance inflammatory responses [233], this hypercoagulable state may also contribute to the increased CVD risk.

Although abovementioned evidence strongly suggests that acromegaly is characterized by a pro-inflammatory state, direct evidence of the relation between acromegaly, inflammation and CVD has not been provided yet. In addition, heterogeneity regarding treatment status, comorbidities as hypertension and insulin resistance, and analytical methods likely contribute to the conflicting results that have been reported previously. Moreover, in vivo findings are not always directly comparable to the ex vivo models, given the difference in or absence of circulating GH, IGF-1, IGFBP and many other interacting factors (adipokines, insulin levels, and adhesion molecules) ex vivo. In addition, cellular signaling, paracrine and autocrine processes could also explain differences between the in vivo and the ex vivo situation, for example by downregulation of the GH receptor [234] and increased IGFBP levels [22]. Last, prolonged exposure to GH or IGF-1 excess is not easily achieved ex vivo due to the limited survival of cells and tissues in culture.

Given these uncertainties, some authors suggested alternative explanation for the presence of premature CVD, such as pressure-related arterial and left ventricular stiffening rather than atherosclerotic disease [108, 230]. However, these hypotheses are based on small numbers of observations and are not substantiated by studies discussing the underlying mechanisms.

Acromegaly treatment is associated with an amelioration of CVD risk and inflammatory status. Some pathologic features were (partially) reverted (e.g. abnormal AT mass, insulin resistance) whereas others were not. This is supported by the reported normalized mortality ratio and prevalence of apparent CAD in well-controlled acromegaly patients, but the persistence of CV comorbidities as hypertension, DM and systemic inflammation and thus the persistence of the increased CVD risk.

In order to further elucidate the relation between inflammation and, CVD in acromegaly, both comprehensive mechanistic as well as prospective research in large and homogeneous cohorts of patients with regard to metabolic features, comorbidities and treatment status, is needed.

## Conclusions

To conclude, the effects of GH and especially IGF-1 on the immune system are versatile, and many factors impact on their cumulative effects on systemic inflammation and CVD. Inflammatory cell-mediated processes also impact on endothelial dysfunction, atherogenesis, (vascular) oxidative stress and metabolic homeostasis and vice versa. Although an independent effect of GH/IGF-1 excess on the vasculature has not been proven, the currently available evidence strongly suggests that both controlled as uncontrolled acromegaly is associated with microvascular damage and endothelial dysfunction, and also pro-inflammatory changes. The mechanisms underlying development of CVD in acromegaly likely involve metabolic disturbances, oxidative stress, and endothelial inflammation and dysfunction. Not surprisingly, these processes are all strongly associated with systemic inflammation and respond to GH/IGF-1 normalizing treatment. This implies that GH/IGF-1, inflammation and CVD are closely, but complexly, intertwined in patients with acromegaly.
